# Transanal small bowel evisceration secondary to rectal perforation in a patient with chronic uterine prolapse: A case report

**DOI:** 10.1016/j.ijscr.2025.111874

**Published:** 2025-08-28

**Authors:** Prakash Shah, Utsav Yadav, Arpan Niroula, Amrit Rijal

**Affiliations:** a Department of Surgery, Nobel Medical College and Teaching Hospital, Biratnagar, Nepal; b Nobel Medical College and Teaching Hospital, Biratnagar, Nepal

**Keywords:** Case report, Evisceration, Perforation, Small bowel, Uterine prolapse

## Abstract

**Introduction:**

Transanal evisceration of small bowel through a rectal perforation is a rare surgical presentation, often associated with rectal prolapse. Its occurrence with uterine prolapse is an uncommon finding.

**Case presentation:**

We report a case of a 50-year-old female with untreated third-degree uterine prolapse and COPD, who presented with transanal evisceration of approximately 100 cm of small bowel following coughing episodes. There was no history of rectal prolapse, trauma, or constipation. After ICU stabilization for sepsis, COPD exacerbation, and diabetic ketoacidosis, she underwent exploratory laparotomy, primary rectal repair, and diverting loop ileostomy. Postoperative recovery was uneventful.

**Discussion:**

Our case adds further evidence to the limited body of literature suggesting a rare complication of chronic uterine prolapse leading to spontaneous rectal perforation. The rise in intra-abdominal pressure from coughing likely triggered the event.

**Conclusion:**

Uterine prolapse may independently predispose to rectal perforation and bowel evisceration. Early recognition and timely surgical intervention are key to favorable outcomes.

## Highlights

• Rare case of transanal small bowel evisceration in a patient with chronic uterine prolapse.
• Chronic uterine prolapse may cause traction-induced rectal wall weakening.
• Cough induced intra-abdominal pressure rise triggered spontaneous rectal perforation.
• Primary rectal repair with ileostomy ensured recovery, demonstrating the effectiveness of damage control surgery.
• Coordinated multidisciplinary care enabled successful treatment despite critical comorbidities.

## 1 Introduction

Transanal evisceration of small bowel through a rectal perforation is a rare and life-threatening surgical emergency with mortality as high as 42.3 % [1]. The earliest documented case was reported by Benjamin Brodie in 1827, and to date, only around 100 cases of transanal evisceration of small bowel have been reported in the literature out of which only two cases were reported in background of uterine prolapse [[2], [3], [4], [5], [6]]. A review of the literature suggests that rectal prolapse is an etiology in the majority of patients [[7], [8], [9]]. However this case is significant due to the occurrence of transanal bowel evisceration in a multiparous women with a long-standing third-degree uterine prolapse. We report a case of a 50-year-old female with chronic uterine prolapse complicated by rectal perforation with transanal evisceration of small bowel following episodes of cough. This case has been reported in line with the SCARE criteria [10].

## 2 Case presentation

**Fig. 1:**
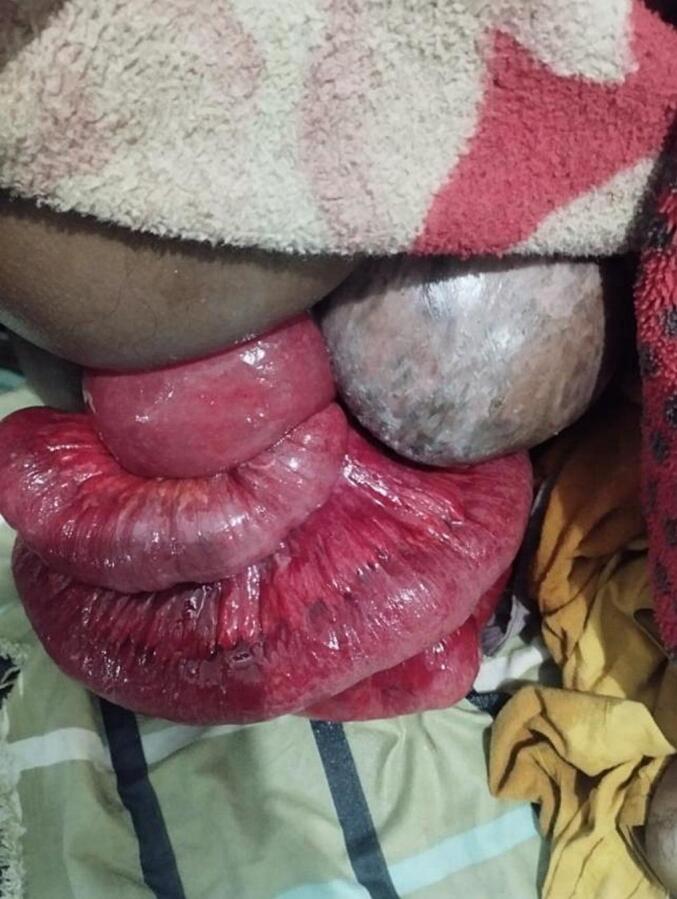
Transanal evisceration of small bowel and prolapsed uterus at presentation.

In March 2025, a 50-year-old female was brought to the Emergency Department by an ambulance, presenting with abdominal pain, spontaneous protrusion of mass through the anus following episodes of cough, multiple episodes of vomiting, and shortness of breath (Fig. 1). She had a known history of chronic obstructive pulmonary disease (COPD) and type 2 diabetes mellitus, both of which were being medically managed. Additionally, she had been living with an untreated third-degree uterine prolapse for the past eight years. She reported no history of trauma, rectal prolapse, chronic constipation, or rectal bleeding.

**Fig. 2:**
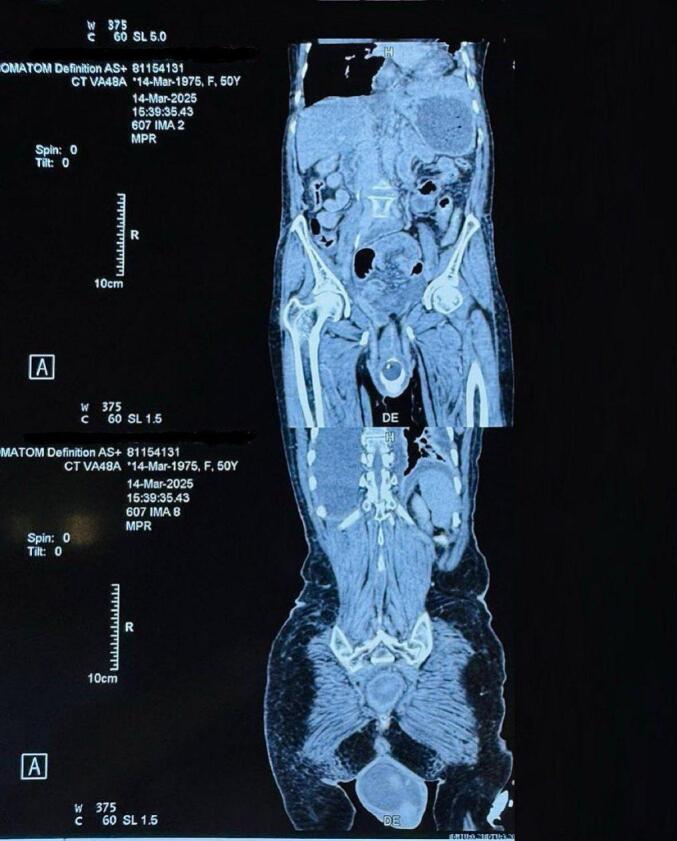
MRI image showing rectal perforation and uterine prolapse.

On arrival, she was alert and febrile. Her vital signs included a blood pressure of 130/70 mmHg, a pulse rate of 80 beats per minute, a respiratory rate of 22 breaths per minute, and an oxygen saturation of 92 % on room air. Chest auscultation revealed decreased air entry bilaterally. Abdominal examination revealed a soft, non-distended but tender abdomen. Perineal examination identified approximately 100 cm of small bowel loops eviscerated through the anus, appearing edematous and congested, along with associated third-degree uterine prolapse. Laboratory results showed hypokalemia (3.4 mEq/dL), a random blood glucose of 305 mg/dL, diabetic ketoacidosis, and leukocytosis with a white blood cell count of 30,000/mm^3^. All other laboratory parameters were within normal limits. Radiological imaging, including a pelvic MRI, was performed. It confirmed the presence of small bowel evisceration through rectum (Fig. 2).

**Fig. 3:**
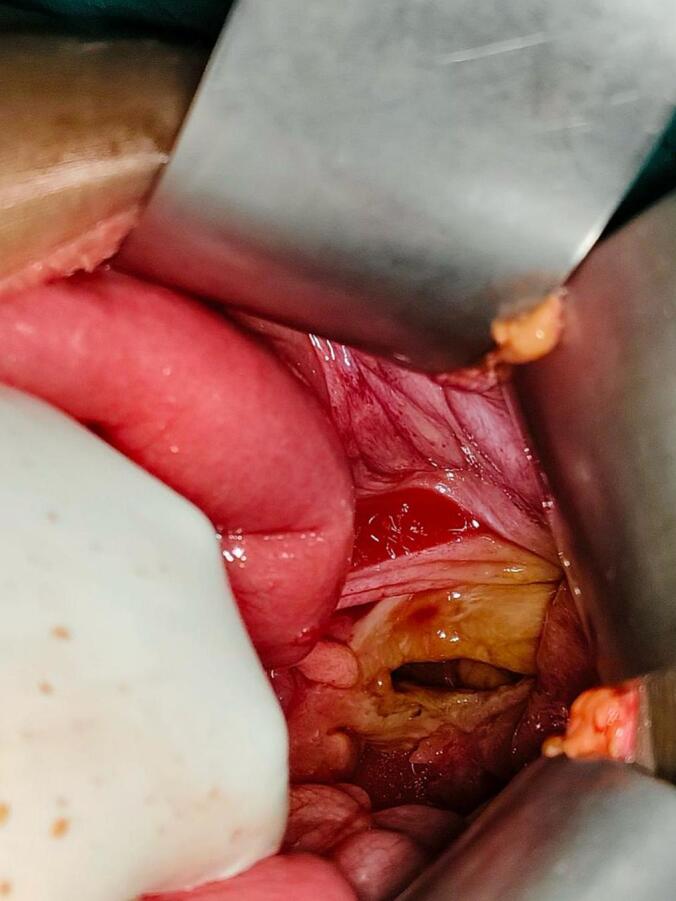
Intraoperative photo of the site of rectal perforation.

Given the acute COPD exacerbation and signs of sepsis, the patient was shifted to the Intensive Care Unit for stabilization. The eviscerated bowel was gently reduced transanally. Medical management was initiated for COPD, sepsis, hypokalemia and diabetic ketoacidosis. Once stabilized, she underwent an exploratory laparotomy under general anesthesia. Approximately 100 cm of edematous ileal loops were reduced into the abdominal cavity through a 5 cm longitudinal perforation in the anterior upper rectum (Fig. 3). Due to presence of fecal contamination, the peritoneal cavity was thoroughly irrigated. The bowel was viable, and a primary repair of the rectal perforation was performed using PDS 3–0 suture. A diverting loop ileostomy and pelvic drain were placed, and the abdomen was closed in layers. Postoperative recovery was uneventful, the stoma was functioning normally and the patient was discharged in stable condition on the seventh day after surgery. At follow-up, the surgical wound had healed without signs of infection, and the patient's nutritional status was good. She reported no new complaints. Definitive management of the underlying uterine prolapse was planned after initial surgical recovery.

### 2.1 Timeline

**Table:** 

Day	Event
**Day 0**	Patient presented with abdominal pain, vomiting, shortness of breath, and bowel evisceration through the anus after coughing. Known COPD, diabetes, and uterine prolapse.
**Day 0**	Vitals stable but febrile. Eviscerated 100 cm of small bowel noted. Labs showed DKA, leukocytosis, and hypokalemia. MRI confirmed rectal perforation.
**Day 0–1**	Admitted to ICU for stabilization. Medical management of DKA, COPD exacerbation, and sepsis. Bowel reduced transanally.
**Day 2**	Exploratory laparotomy performed. Rectal perforation (5 cm) identified and primarily repaired. Viable bowel reduced. Diverting ileostomy and pelvic drain placed.
**Post-op days 1–6**	Recovery uneventful. Stoma functional. Continued supportive care.
**Day 7**	Patient discharged in stable condition.
**Follow-up**	Wound healed, good nutritional status. No new complaints. Prolapse surgery planned.

## 3 Discussion

Uterine prolapse, although much less commonly reported, has been suggested as a contributing factor to transanal evisceration in very few cases. Our review of the literature revealed only two prior reports describing third-degree chronic uterine prolapse associated with rectal perforation and transanal small bowel evisceration [5,6]. The majority of these cases are associated with predisposing factors such as chronic rectal prolapse, severe constipation, trauma, or perineal surgery [[7], [8], [9]]. Our case emphasize on the fact that uterine prolapse can also lead to presentation of rectal perforation with transanal bowel evisceration as its terminal complication.

The exact pathophysiology behind this condition remains unclear. One proposed mechanism is that continuous traction exerted by the prolapsed uterus on the anterior rectosigmoid wall created tearing forces, which eventually led to rupture of the bowel wall under increased intra-abdominal pressure [5,6]. In elderly patients, chronic uterine prolapse may result in ischemia and mucosal atrophy of adjacent pelvic structures, thereby increasing the risk of spontaneous rectal perforation [11]. Uterine prolapse is not an isolated condition; rather, it reflects generalized pelvic floor dysfunction, which predisposes patients to concurrent prolapse and potential injury of adjacent organs, including the rectum [12]. Sudden increase in intra-abdominal pressure can lead to rupture especially in weakened area of rectosigmoid [13]. In our case, the patient was an elderly multiparous woman which is one of the risk factor contributing to pelvic floor weakening and the development of uterine prolapse. Over time, progressive weakening of the anterior rectal wall occurred, and repeated episodes of coughing due to underlying COPD likely caused a sudden increase in intra-abdominal pressure, resulting in rupture of the already compromised rectal wall which leads to transanal evisceration of small bowel.

Our case adds further evidence to the limited body of literature suggesting that uterine prolapse, especially when left untreated, may be an independent risk factor for rectal wall weakening and subsequent perforation. Notably, our patient had no history of trauma, rectal prolapse, or other commonly associated factors, reinforcing the likely causative role of uterine prolapse compounded by chronic pressure changes from COPD.

Management lacks established guidelines and typically follows the general principles of management of abdominal trauma. Timely intervention is critical, as delays may result in strangulation or ischemia of the eviscerated bowel [14]. In our case, the patient was initially stabilized from acute COPD exacerbation, sepsis, hypokalemia and diabetic ketoacidosis. Management of the case involved exploratory laparotomy, reduction of viable small bowel, primary repair of the rectal perforation, and formation of a diverting loop ileostomy. The choice to proceed with primary repair was made due to the presence of a single, non-destructive injury, accompanied by a well-vascularized, healthy mesentery without any tears. This approach aligns with the literature favoring immediate resuscitation, surgical exploration, and bowel viability assessment [7,14]. In previously reportedly similar literature intestine was nonviable so it was resected and primary repair of rectosigmoid was done along with sigmoid loop colostomy following with patient recovered without complication [13]. Alternative approaches such as Hartmann's procedure, Thiersch repair, laparoscopic repair or transanal techniques have been reported but are case-dependent [7,15]. Our approach, balancing patient condition and contamination risk, yielded favorable outcomes. If left untreated, this condition has 100 % mortality; however, timely and appropriate treatment significantly reduces the risk of mortality [16].

## 4 Conclusion

Transanal evisceration of the small bowel through a rectal perforation is a rare surgical presentation. This case highlights the potential of untreated uterine prolapse to cause rare complication in the form of spontaneous rectal perforation and small bowel evisceration. Early treatment of uterine prolapse is necessary to prevent such complication. Clinicians should maintain a high index of suspicion in females with uterine prolapse and acute anorectal symptoms. Early recognition, prompt stabilization, and timely surgical intervention are key to favorable outcomes during such surgical emergency. Also adequate treatment of COPD should be considered in such situations.

## CRediT authorship contribution statement

Amrit Rijal: Conceptualization, literature review, drafting of the manuscript, and final editing.
Arpan Niroula: Collection and analysis of clinical data, literature review, drafting of the manuscript.
Dr. Utsav Yadav: Supervision, Final approval of the manuscript.
Dr. Prakash Shah: Surgical management of the case, critical revision of the manuscript.

## Informed consent

Written informed consent was obtained from the patient for publication of this case report and accompanying images.

## Ethical approval

Not applicable for single case reports at our institution.

## Guarantor

Dr. Prakash Shah.

## Patient perspective

I had uterine prolapse for many years, but did not realize it required treatment. When I suddenly felt pain and something came out from my rectum, I was very terrified. The doctors explained everything clearly, treated me with great care, and I am grateful for the surgery that saved my life. Now I know how important it is to take health problems seriously and get checked in time.

## Research registration number

1. Name of the registry: Not applicable
2. Unique identifying number or registration ID: Not applicable
3. Hyperlink to your specific registration: Not applicable.

## Funding

No funding was received for this study.

## Declaration of competing interest

The authors declare no conflicts of interest.
